# A comparative study of flaxseed gum effect on *Lactobacillus acidophilus* genes expression, and textural, sensory, structural, and microbiological properties of synbiotic Iranian white cheese

**DOI:** 10.1038/s41598-025-17819-z

**Published:** 2025-08-29

**Authors:** Mahya Soltani, Ehsan Sadeghi, Hooman Shirvani

**Affiliations:** 1 Student Research Committee, Department of Food Science and Technology, School of Nutrition Sciences and Food Technology, Kermanshah University of Medical Sciences, Kermanshah, Iran; 2 Department of Food Science and Technology, School of Nutrition Sciences and Food Technology, Research Center for Environmental Determinants of Health (RCEDH), Health Institute, Kermanshah University of Medical Sciences, Kermanshah, Iran; 3 Nutrition Sciences and Food Technology Research Center, Health Institute, Kermanshah University of Medical Sciences, Kermanshah, Iran

**Keywords:** Gene expression, *Lactobacillus acidophilus*, Flaxseed gum, Iranian white cheese, Synbiotic, Biotechnology, Microbiology

## Abstract

Flaxseed gum (FG), a natural polysaccharide with prebiotic potential, may enhance probiotic functionality by modulating bacterial gene expression and improving viability in synbiotic dairy matrices. This study evaluated the effect of FG (2.5% and 5%) on *Lactobacillus acidophilus* gene expression, viability, sensory properties, texture, and microstructure in Iranian white cheese. Four functional genes were selected for their roles in amino acid biosynthesis, stress adaptation, metabolic regulation, and cell wall integrity. Gene expression was assessed on days 15 and 60 using real-time PCR, while bacterial viability was measured by PMA-qPCR and plate count methods. Significant differences in relative gene expression were observed across all treatments and both time points (day 15 and day 60), with distinct fold-change values for each gene. All gene expressions peaked on day 15 and declined by day 60, possibly due to adaptive stress responses or diminishing FG effect. On day 60, the FG 5% treatment exhibited the highest *L. acidophilus* viability (1.55 × 10^7^ CFU/g), outperforming both the control and other treatments, and highlighting its superior protective effect during cheese ripening. Sensory analysis revealed FG 5% had the highest general acceptance score, with textural and structural parameters (springiness, chewiness, and porosity) comparable to or improved over control. SEM and ImageJ analyses confirmed enhanced matrix cohesion, reduced porosity (7%), and finer fat dispersion (10 μm) in FG 5%. These findings show that FG may support probiotic viability and cheese quality, offering new insight into the molecular basis of probiotic survival and a promising strategy for developing functional dairy products.

## Introduction

Cheese is one of the most widely consumed dairy products worldwide, available in diverse varieties differing in morphology, texture, and sensory properties^1,2^. Among these, Iranian white cheese, commonly known as breakfast cheese, holds a prominent place in Iranian cuisine. It shares similarities in texture with Turkish white cheese and Feta; however, notable differences exist in its production process compared to Feta cheese^3^. Given its widespread consumption, Iranian white cheese presents considerable potential for development as a functional food. Incorporating probiotics and prebiotics into this cheese may offer promising benefits for enhancing public health^4^.

Probiotic dairy products, especially those formulated with fermented milk, are manufactured at large scales globally, owing to the greater compatibility of dairy matrices with probiotic microbes over other food carriers^5^. *L. acidophilus* is among the most widely used probiotics in the dairy industry. This well-established probiotic is commonly added to milk, yogurt, cheese, infant formula, and dietary supplements. Numerous studies have confirmed its safety and effectiveness, particularly in alleviating gastrointestinal disorders such as diarrhea. Moreover, the inclusion of prebiotics can further enhance the activity and health benefits of probiotic bacteria^6,7^.

Flaxseed (*Linum usitatissimum* L.) gum is a natural heteropolysaccharide extracted from the outer mucilaginous layer of flaxseeds, constituting about 8% of the seed weight. It contains neutral and acidic monosaccharides and shares properties with gums like guar, arabic, tragacanth, and carrageenan^8–10^. Known for its prebiotic effects and texture-modifying properties, flaxseed gum (FG) can stimulate probiotic bacterial growth and has been associated with health benefits including lowering blood cholesterol and triglycerides and improving gastrointestinal and cardiovascular conditions^11^. Incorporating FG into Iranian white cheese may enhance texture, support probiotic viability, and influence the expression of genes related to probiotic functions.

Gene expression is of critical importance in differentiation and functional characteristics of living organisms. In foods, an analysis of gene expression offers valuable information on microbial physiology, particular functionality, and association with sensory and structural food properties^12,13^. Investigating the expression of probiotic genes can facilitate the development of functional dairy products and establish links between bacterial growth and health-promoting attributes^14^.

This project discusses prominent genes that occur in probiotics, which include *Aspartate kinase*, *SOS* response-related peptidase, *ROK* family protein, and *LytR-CpsA-Psr* (*LCP*). Aspartate kinase enables the initial step in biosynthesis of fundamental amino acids (lysine, threonine, isoleucine, and methionine). Control of its activity by a feedback inhibition pathway through lysine promotes a balance production of amino acids^15–18^. The *SOS* response pathway, which is induced by DNA damage, is also significant in repairing DNA, cell cycle regulation, and genome stability. It enhances the viability of probiotic bacteria like *L. acidophilus*, thus their probiotic characteristics^19–21^.

The *ROK* family of proteins functions as repressors and kinases, effectively suppressing pathogenic bacteria such as *Escherichia coli*, other species of *Staphylococcus*, and *Haemophilus* influenzae. Such microorganisms are commonly associated with gastrointestinal symptoms and opportunistic infections, particularly among those who have weakened immune systems and in children^22–26^. Meanwhile, *LCP* proteins assist in ensuring the integrity of bacterial cell walls during stressful environments, including beta-lactam antibiotic resistance, thereby enhancing the viability and survival of probiotics^27,28^.

Although other genes such as those related to adhesion to intestinal epithelial cells, bile salt tolerance, or acid resistance are also important for defining probiotic functionality and could provide valuable insights into bacterial persistence within the gastrointestinal tract, our study prioritized genes representing the three fundamental domains of metabolism, structural integrity, and stress response. These genes not only contribute to the survival of microorganisms under the adverse conditions of the cheese matrix but also play a direct role in enhancing metabolic activity, maintaining cell wall stability, and supporting adaptation to environmental fluctuations. Therefore, their analysis offers a more integrated and comprehensive view of *L. acidophilus* adaptation and performance during cheese ripening, while simultaneously providing a mechanistic basis for understanding the interaction between prebiotics and the molecular responses of probiotics in dairy products. This approach, in addition to the novelty of the selected targets, lays the foundation for future studies on other functional genes related to host–microbe interactions and gastrointestinal persistence.

The objective of this study was to elucidate the effects of FG supplementation at 2.5% and 5% concentrations on the expression of key functional genes involved in stress response, metabolism, and cell wall integrity of L. acidophilus in Iranian white cheese. Furthermore, the research aimed to assess how FG influences probiotic viability, sensory attributes, textural properties, and microstructural characteristics throughout cheese ripening. By integrating molecular gene expression analysis with physicochemical and sensory evaluations, this study seeks to provide comprehensive insights into the role of FG in enhancing probiotic stability and improving the overall quality of synbiotic dairy products, thereby contributing to the development of functional foods with optimized health benefits and consumer acceptance.

## Materials and methods

### Materials

Standard milk with 1.5% fat content was obtained from a local dairy factory for cheese production. Flaxseed was sourced from the Kermanshah Agricultural Research Center. The probiotic strain *L. acidophilus* (ATCC 53544) was provided by the Research Organization for Science and Technology of Iran (Center for Industrial Microorganisms Collection). Other chemicals and reagents used in this study including trisodium citrate, Tris buffer, EDTA, calcium chloride, DEPC, D-sorbitol, nalidixic acid, neomycin sulfate, lithium chloride, paramomycin, propidium monoazide (PMA), phosphate-buffered saline tablets (Sigma-Aldrich, USA), agarose, boric acid, MRS agar medium, peptone water, gas packs (Merck, Germany), cheese starter culture includes *Lactococcus lactis* and *Lactococcus cremoris* (Chr. Hansen, Denmark), nucleic acid extraction kits (Kiagene, Iran), loading buffer, DNA ladders, Safe Stain, primers (Sinnaclone, Iran), SYBR Green Master Mix/Rox kit, and DNase I enzyme (Ampliqon, Denmark) were purchased from standard commercial suppliers.

### Flaxseed gum extraction

FG was extracted by soaking 500 g of flaxseeds in 4 L of distilled water at 85–90 °C and with continuous stirring for 24 h. The optimal pH for extraction was 5.6 to 7. The mucilage was separated from the seeds using a mesh cloth with medium-sized pores. The collected mucilage was then centrifuged at 2700 rpm for 25 min at 0 °C to remove insoluble particles. Subsequently, the supernatant was precipitated by adding 100% ethanol, followed by centrifugation at 3000 rpm for 25 min at 0 °C to isolate the polysaccharide. The precipitated FG was washed with distilled water and stored at − 20 °C until further use^29–31^.

### Bacterial activation

A 100 µL aliquot of *L. acidophilus* suspension was inoculated into 10 mL of MRS broth and incubated at 37 °C for 18 h. The subculturing was repeated two to three times in fresh MRS broth until the bacterial population reached approximately 10^8^–10^9^ CFU/mL. Cells in the logarithmic growth phase were then harvested by centrifugation at 3000 rpm for 5 min at 4 °C, followed by two successive washes with 0.1% peptone water to ensure purification^32^.

### Cheese production

Laboratory-scale cheese samples were produced using a Manizan dairy factory pilot system. Initially, the microbial and physicochemical properties (pH, fat content, protein content, total solids, titratable acidity, ash content, freezing point, and density) of the raw milk were evaluated before transferring 4 L into the equipment. The fat content was adjusted to 1.5%, and essential additives for cheese production, including calcium chloride (0.02 g/L) and FG (at specified concentrations), were incorporated into the milk^33^. The milk was then pasteurized and cooled to 35 °C. Subsequently, 0.04 g/100 mL of a starter culture (*Lactococcus lactis* and *Lactococcus cremoris*) and 1 mL of a *L. acidophilus* probiotic suspension were added. The mixture was incubated at 35 °C for 55 min to allow bacterial growth. The curds were cut into 1 cm cubes and left for 10 min to allow whey drainage. After that, they were gently stirred for 10 min to improve whey expulsion, and then pressed for 2.5 h to remove excess moisture^34,35^. The cheese was cut into pieces measuring 6 × 6 × 4 cm and soaked in 20% brine for 8 h. It was then transferred to 8% brine, vacuum-sealed in pasteurized bags, and underwent a 15-day ripening period at 22 °C, known as the early (green) storage phase. After ripening, the cheese was stored at 4 °C for 60 days until it was ready to be consumed^36,37^.

To enable accurate comparison of parameters, three types of cheese were produced: (1) control cheese containing only the starter culture, (2) probiotic cheese with starter and *L. acidophilus* (PSL), and (3) synbiotic cheese with starter, *L. acidophilus*, and FG at concentrations of 2.5% (SSL-FG 2.5%) and 5% (SSL-FG 5%).

### Gene expression of *L. acidophilus*

The molecular response of *L. acidophilus* to the addition of FG in Iranian white cheese was analyzed by assessing the expression of *Aspartate kinase*, *SOS response-associated peptidase*, *ROK family*, and *LCP (LytR-CpsA-Psr)* genes. Gene expression was evaluated at two time points: the end of the green storage period (The 15th day) and market entry (The 60th day).

To ensure RNA integrity and prevent degradation, all tools needed for RNA extraction were sterilized to inhibit Rnases^38^. For total RNA extraction, 2 g of cheese sample was homogenized with 8 mL of a 2% (w/v) trisodium citrate solution, followed by centrifugation (4 °C/6000 rpm/5 min). The supernatant was discarded, and RNA extraction continued using the RNA kit^39^. The quantity, and concentration of RNA were determined by NanoDrop device (model 2000 C, Thermo Fisher Scientific, USA). The quality of the total RNA extracted was then verified through 1% agarose gel electrophoresis^39,40^.

To eliminate any genomic DNA contamination from the RNA samples, all RNA samples were treated with a DNase kit. Subsequently, cDNA synthesis was conducted using a Reverse Transcription Kit. The necessary components were added to the appropriate tube from the cDNA synthesis kit and incubated at 55 °C for 60 min. The samples were then incubated at 95 °C for 5 min in a water bath, followed by cooling on ice and storing at − 80 °C^40,41^. Real-time PCR was performed using a SYBR Green master mix/Rox kit. To evaluate the efficiency of each primer pair, a mix of all treatments and replicates of the synthesized cDNA was prepared. Various dilution factors were tested for each primer pair. After determining the optimal concentration of primer and cDNA, gene expression was assessed using Real-time PCR (StepOne, Applied Biosystems, USA)^41,42^.

For each sample, individual reaction volumes were prepared in separate wells of the MicroAmp Fast Optical 48-Well Reaction Plate, and PCR was performed under the following conditions: (1) Predenaturation: 95 °C for 5 min, (2) PCR reaction: 95 °C for 30 s, 60 °C for 30 s, 72 °C for 30 s for 40 cycles, 3), final extension 72 °C for 5 min, 4) melting curve analysis: 95 °C for 15 s, 60 °C for 1 min, 95 °C for 15 s (0.5 °C), followed by cooling to 4 °C^43,44^.

In this study, *Rec A* gene was used as the reference^45^. Primer design was performed using Oligo7 and Primer3 software, followed by validation with Primer-BLAST for final confirmation. The targeted genes and primer characteristics are presented in Table 1. Relative gene expression levels were calculated using the 2^^−ΔΔCT^ method, utilizing the melting temperatures determined for each primer^46^.

### Viability assays of *L. acidophilus*

#### Plate count

The viability of *L. acidophilus* probiotic bacteria in each cheese sample was assessed at different ripening stages (days 0, 15, 30, and 60)^47^. For this purpose, 10 g of homogenized cheese was suspended in 90 mL of peptone water and then serially diluted down to 10^−9^. 1 ml from each dilution was plated on MRS agar using the pour plate technique^48^. The plates were incubated under anaerobic conditions at 37 °C for 72 h, after which bacterial colonies were counted to determine viability^49^. To selectively promote the growth of *L. acidophilus*, D-sorbitol (10 mL of a 10% solution per 90 mL of MRS agar) was added to the medium^50,51^.

#### PMA-real-time PCR

To quantify *L. acidophilus* using Real-Time PCR, the bacteria were initially cultured in MRS medium and incubated at 37 °C for 24 h. This process was repeated three times to ensure optimal bacterial growth^52^. Subsequently, serial dilutions (V1–V12) of the original culture were prepared. Cheese samples (5 g) were homogenized in 45 mL of phosphate buffer and centrifuged at 6000 rpm for 10 min. The supernatant was discarded, and the pellet was resuspended in ultrapure water^53^.

A 20 mM PMA stock solution (1 mg PMA in 20% dimethyl sulfoxide) was prepared and stored at − 20 °C. PMA was added to the bacterial dilutions and homogenized cheese samples to a final concentration of 50 µM. Samples were incubated in the dark for 5 min, followed by exposure to halogen light (500 W) for 15 min to cross-link free DNA. Afterward, samples were centrifuged and subjected to DNA extraction^54^.

DNA extraction was performed using a commercial kit following the manufacturer’s protocol^55^. Briefly, 180 µL of lysing enzyme buffer was added to the bacterial pellet, followed by 20 µL of proteinase K, and incubated at 56 °C for 30 min. An additional 200 µL of lysing enzyme buffer was then added before proceeding with DNA precipitation and washing steps. Finally, the DNA was recovered by transferring the mixture to a sterile tube and centrifuging at 6000 rpm for one minute. Real-Time PCR amplification was carried out as previously described, using 16 S rRNA as the target gene for bacterial quantification and RecA as the reference gene (Table 1). The total reaction volume was 20 µL, and cycle threshold (CT) values were determined in triplicate using the SYBR Green master mix/Rox kit. A standard curve was generated based on CT values from serial dilutions, enabling quantification of *L. acidophilus* at days 0, 15, 30, and 60 of cheese ripening.

**Table 1:** Characteristics of primers designed for *L. acidophilus* (ATCC 53544).

Description	Sequence	Length	Tm	GC%	Product length
*Aspartate kinase*	F: 3’ GCCTTGAATGTAGCCATGCG 5’	20	59.8	50	251
	R: 5’ GTGGTTTCTGCCCCTGGTAA 3’	20	59.8	55	
*SOS response-associated peptidase*	F: 3’ GGCTACCCCAGTCCAGTAGA 5’	20	60.0	60	347
	R: 5’ TGAAAAAGCCAACGCCGAAG 3’	20	59.9	50	
*ROK family protein*	F: 3’ ACTTGGACCAGCTGCCATAC 5’	20	60.0	55	160
	R: 5’ TGTTGGGGCTGGTATCGTTC 3’	20	60.2	55	
*LCP family protein*	F: 3’ ACGCGATACCACGCAAAATA 5’	20	58.3	45	157
	R: 5’ ACGTCCTTTCCAGCTACGTC 3’	20	59.7	55	
*RecA*	F: 3’TCCAAGTGCCTCGGCATAAG5’	20	60.1	55	246
	R: 5’ATGCGCATGGGTGAAAAAGC3’	20	60.3	50	
16 S r RNA	F: 3’TGCCGTAAACGATGAGTGCT 5’	20	60.5	50	365
	R: 5’GTTTGTCACCGGCAGTCTCA 3’	20	60.5	55	

F: forward, R: reverse.

### Final evaluation for entering the consumer market

#### Sensory properties

Sensory evaluation of the cheese samples was conducted in the Quality Control Laboratory of the Faculty of Nutrition Sciences and Food Industries using a five-point hedonic scale ranging from “very good” to “very poor.” Fifteen trained panelists, aged 18 to 50 years old and regular consumers of dairy products, participated in the study. The assessment took place at the end of the ripening period (day 60), focusing on flavor, texture, color, and overall acceptability^56^. Prior to evaluation, panelists received training on the sensory testing protocol. Each participant was provided with a coded checklist and 30 g of each cheese sample, served in white containers with randomized order. The evaluations were carried out under controlled lighting and room temperature to ensure consistency. To prevent sensory fatigue and cross-sample contamination, panelists rinsed their mouths with water between samples. The acceptance scores were analyzed to identify the most preferred cheese formulations^57,58^.

#### Texture properties

Texture analysis was conducted on cheese samples at the conclusion of the 60-day ripening period. Each sample was cut into 2 × 2 × 2 cm cubes and evaluated for essential textural characteristics, such as adhesiveness, cohesiveness, stringiness, and chewiness. Before testing, the samples were allowed to stabilize at 25 °C for 30 min. The assessment was carried out using a TX-700 N texture analyzer from Lamy, France, with an aluminum probe that compressed the samples at a controlled rate until they reached 50% deformation of their diameter. To ensure accuracy, each measurement was repeated three times per sample^47,59^.

#### Scanning electron microscopy (SEM)

To evaluate the morphological characteristics of cheese samples at the end of the ripening period (day 60), each sample was sliced into approximately 0.5 cm pieces and fixed in 2.5% (w/v) glutaraldehyde solution for 3 h. After fixation, samples were rinsed with distilled water and dehydrated through a graded ethanol series (40%, 55%, 70%, 85%, 90%, and 96%), with each step lasting 30 min. Subsequently, lipid extraction was performed by immersing the samples in chloroform three times for 10 min each. The processed samples were then stored in ethanol at 4 °C until further analysis. Prior to scanning electron microscopy (SEM), cheese fragments were rapidly frozen using liquid nitrogen and fractured into smaller pieces (~ 1 mm). Finally, samples were mounted on aluminum stubs and observed under SEM (EVO 40, Carl Zeiss, Germany) at various magnifications to examine their microstructural features^60,61^.

### Statistical analysis

Gene expression data were analyzed using a two-way factorial ANOVA within a completely randomized design, considering two independent factors: cheese type and time. The assumptions of ANOVA (normality and homogeneity of variances) were verified using the Shapiro Wilk and Levene’s tests, respectively. Mean comparisons were subsequently performed using Duncan’s multiple range test at a significance level of 1%. Sensory evaluation, texture, and structural features data were analyzed using a one-way factorial ANOVA within a completely randomized design (*P* ≤ 0.01), with the same assumption checks and Duncan’s post hoc test. Graphical visualizations were performed using GraphPad Prism 8 and XLSTAT. For integrated analysis of sensory and textural attributes, two-way cluster analysis was applied to classify samples and traits, simultaneously producing heatmap visualizations. This analysis was conducted using ClassVise software.

## Results and discussion

### Gene expression

In the present study, FG was incorporated into Iranian white cheese at concentrations of 2.5% and 5% to evaluate its modulatory effects on the expression of four functional genes in *L. acidophilus*: *Aspartate kinase*, *SOS response-related peptidase*, *ROK family protein*, and *LytR-CpsA-Psr (LCP)*. These genes were selected based on their roles in amino acid biosynthesis, stress response, carbohydrate metabolism, and cell wall stability, which are critical for the survival and functionality of probiotic strains in food matrices. Gene expression levels were analyzed at two time points (days 15 and 60 of ripening), and the data obtained were subjected to analysis of variance.

The statistical analysis demonstrated that both the type of cheese and the ripening time had a highly significant effect on gene expression (*p* < 0.01), as did their interaction. The mean square values for the genes ranged from 8.70 to 12.59, with *Aspartate kinase* showing the highest variance, while *ROK family protein* had the lowest. Coefficient of variation (CV%) values were acceptably low (9.08–12.21), and residual error was minimal, indicating that the model was both reliable and statistically robust (Table 2).

**Table 2:** Variance analysis of gene expression during ripening time in cheeses produced.

Source	df	Mean square			
		*Aspartate kinase*	*LCP family protein*	*ROK family protein*	*SOS response-associated peptidase*
Treatments	2	12.59**	10.46**	8.70**	10.71**
Time	1	14.70**	10.90**	7.19**	6.16**
Treatments × Time	2	3.46**	2.48**	1.53**	1.28**
Error	18	0.039	0.020	0.023	0.046
CV%		12.21	9.07	10.54	11.38

df: degrees of freedom, CV: Coefficient of Variation.

**Significance (p < 0.01).

**Fig. 1:**
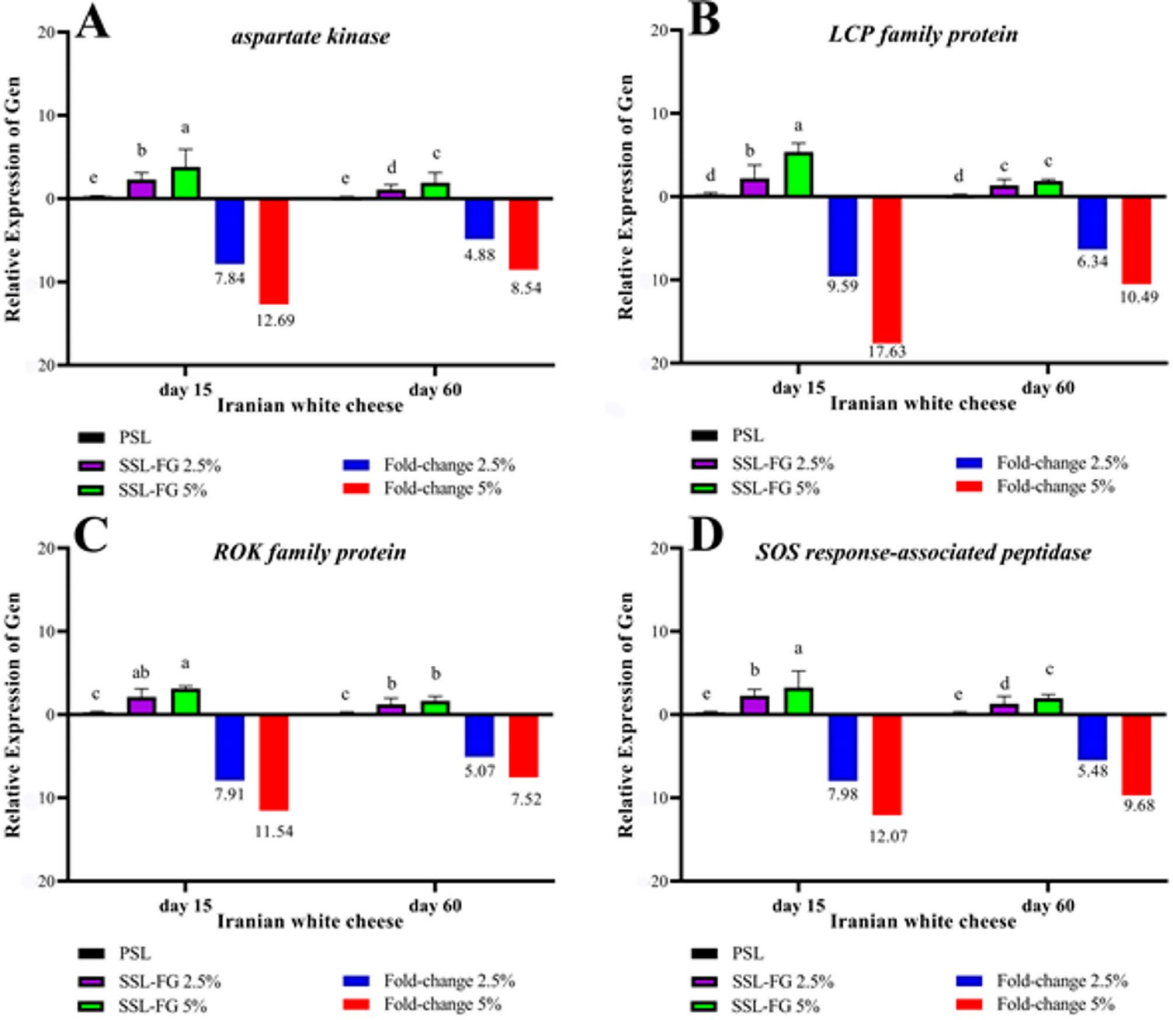
Relative expression levels of the studied genes, normalized to the reference gene *Rec A*, in synbiotic (SSL-FG 5% and SSL-FG 2.5%) and probiotic (PSL) cheeses.

As shown in Fig. 1A, among the genes studied, *Aspartate kinase* exhibited the most notable upregulation, with fold-changes of 12.69 and 7.84 on day 15 in FG 5% and FG 2.5% samples, respectively. Although the expression decreased by day 60 (8.54 and 4.88, respectively), it remained significantly higher than in probiotic-only controls. This enzyme catalyzes the first step in lysine biosynthesis, which not only contributes to the metabolic activity of the bacteria but also potentially enhances the nutritional profile of the cheese. Similar trends have been observed in studies where *Lactobacillus delbrueckii* demonstrated upregulation of *FolE* in response to lactose as a prebiotic. This further confirms the connection between prebiotic compounds and amino acid biosynthesis^62^. The upregulation of such genes may also be indicative of greater metabolic vigor and microbial stability, contributing to improved probiotic viability throughout storage.

Similarly, the expression of the *LytR-CpsA-Psr (LCP)* gene, which plays a role in maintaining the structural integrity of bacterial cell walls, significantly increased under the influence of FG. On day 15, fold-change values were 17.63 and 9.59 for FG 5% and FG 2.5%, respectively. These values decreased to 10.49 and 6.34 by day 60 (Fig. 1B). The increased expression of this gene may help the bacterium resist acidic and osmotic stresses, potentially leading to improved tolerance to gastrointestinal conditions. Similar protective roles have been observed for S-layer associated genes like *SlpX* and *SlpA* in *L. acidophilus* when exposed to salt stress, as their heightened expression enhances structural resilience^63^. This implies that FG may act not only as a metabolic stimulant but also as a structural modulator that helps maintain probiotic integrity during ripening and after ingestion.

The *ROK family protein* gene, which plays roles in sugar metabolism and transcriptional regulation, also exhibited a significant increase in expression during early ripening. On day 15, fold-change values reached 11.54 (FG 5%) and 7.91 (FG 2.5%), reducing to 7.52 and 5.07 on day 60 (Fig. 1C). The early increase in expression suggests that FG provided fermentable substrates that activated carbohydrate-utilizing pathways, enhancing bacterial competitiveness. The functional benefits may extend beyond microbial metabolism; prior studies have associated *ROK* proteins with suppression of pathogens such as *E. coli* and *S. aureus* and the modulation of host immune responses^25,26^. In cheese, such effects are especially valuable in improving microbial safety and probiotic functionality during storage and gastrointestinal transit.

The *SOS response-related peptidase* gene, responsible for DNA repair and cellular resilience under stress, also responded positively to FG supplementation. Expression peaked on day 15, with fold-changes of 12.07 (FG 5%) and 7.98 (FG 2.5%), then declined to 9.68 and 5.48 by day 60 (Fig. 1D). This pattern aligns with the known temporal dynamics of stress-response genes, which are typically activated during early exposure to environmental challenges and decrease as microbial adaptation progresses. The enhanced expression of this gene suggests that FG may help preserve the genomic integrity of probiotics during cheese ripening and storage, thereby improving their industrial stability and long-term viability. Similar patterns have been noted in *L. paracasei* in brined cheeses, where expression of stress-related genes such as *dnaK* and *groES* shifted throughout the ripening period^64^.

The temporal trend observed across all genes elevated expression at day 15 followed by a reduction at day 60 may reflect a combination of metabolic adaptation, substrate depletion, and reduced bioavailability of FG components over time. This trend is consistent with findings from Noutsopoulos et al.^65^where the expression of the *nisA* gene declined with prolonged fermentation. Nonetheless, the sustained gene activity, particularly in the FG 5% group, suggests that the synbiotic matrix successfully supported probiotic function over the full ripening period.

When contextualized within existing literature, the findings of this study contribute novel insights into the molecular-level functionality of *L. acidophilus* in traditional dairy matrices. While previous research has explored probiotic gene expression in cheeses for purposes such as antifungal peptide production, flavor enhancement, or survival in harsh environments, the specific genes analyzed in the present study particularly *Aspartate kinase*, *LCP*, *ROK*, and *SOS-peptidase* have not been previously investigated in Iranian white cheese. The modulation of these genes by FG underscores its potential as a bioactive component in synbiotic formulations. In summary, FG positively influenced the expression of key functional genes in *L. acidophilus*, reflecting improved metabolic activity, stress resilience, and structural integrity of probiotic cells during cheese ripening. These molecular findings not only support the use of FG as a functional ingredient but also highlight its role in enhancing the probiotic quality and health-promoting potential of traditional dairy products.

### Viability assays

**Fig. 2:**
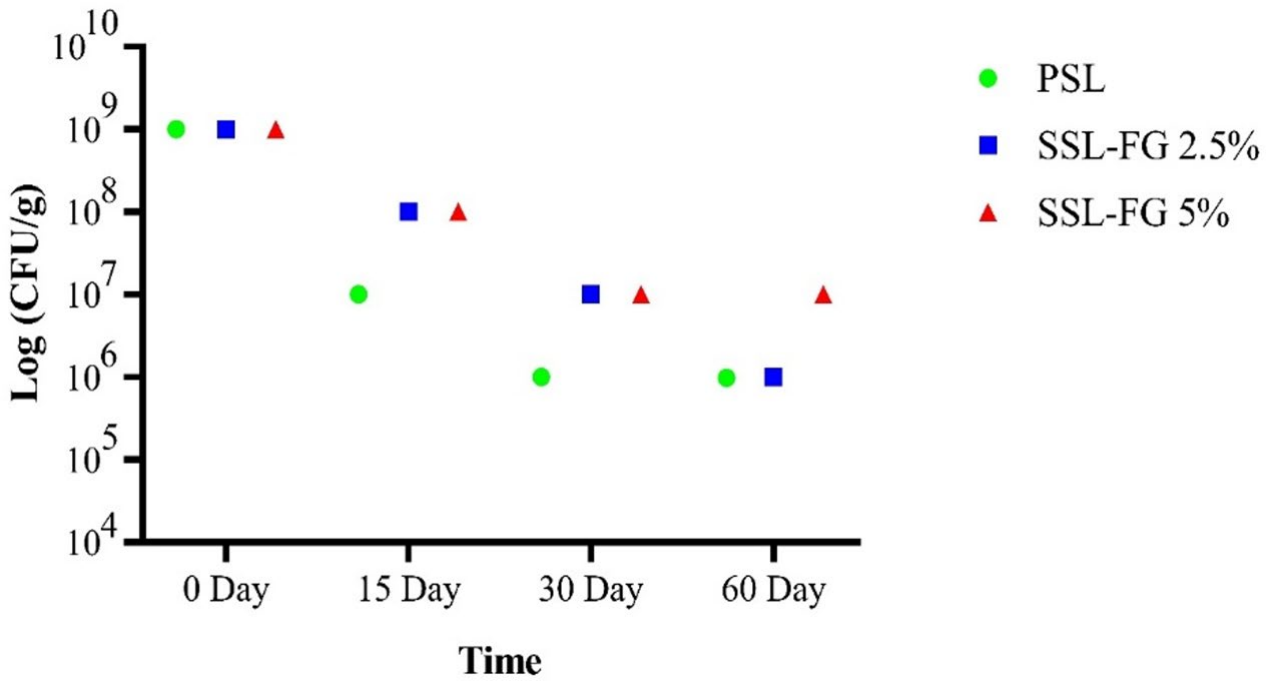
Enumeration of *L. acidophilus* using the plate count on MRS agar.

Microbial enumeration in MRS medium (Fig. 2) revealed that at the initial time point (0th day), all cheese samples exhibited a high probiotic load of approximately 10^9^ CFU/g, confirming the successful inoculation of *L. acidophilus*. However, a gradual decline in bacterial viability was observed during the ripening and storage periods. Notably, after 15 days of green storage, the probiotic count in the PSL (probiotic cheese without FG) sample sharply decreased to 10^7^ CFU/g, whereas synbiotic cheeses supplemented with FG (SSL-FG 2.5% and SSL-FG 5%) maintained higher viability levels of 10^8^ CFU/g. This trend persisted on day 30, with the PSL group further declining to 10^6^ CFU/g, while FG enriched samples sustained counts at 10^7^ CFU/g. At the conclusion of the 60-day ripening period, the SSL-FG 5% sample preserved the highest bacterial population (~ 10^7^ CFU/g), outperforming both the PSL and SSL-FG 2.5% samples, which stabilized at 10^6^ CFU/g. These findings suggest that FG, particularly at a 5% concentration, acts as an effective prebiotic substrate that enhances the stability and survival of probiotic bacteria during cheese maturation, potentially by protecting cells from environmental stresses and nutrient depletion. Similar protective effects of plant-derived gums on probiotic viability have been documented, as exemplified by Alhssan et al.^66^who reported increased survival and growth rates of *L. acidophilus* and *Bifidobacterium lactis* in kefir fortified with gum arabic and flaxseed mucilage.

**Fig. 3:**
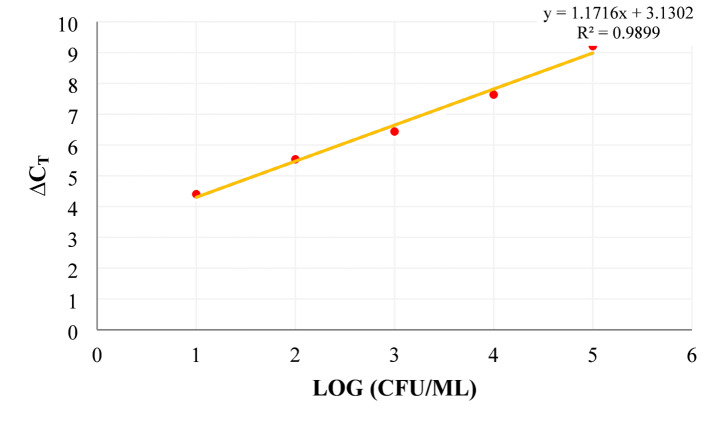
Standard curve obtained from dilution of extracted *L. acidophilus* DNA using the PMA-Real-time PCR method.

Complementing plate counts, quantitative PCR (qPCR) combined with propidium monoazide (PMA) treatment was employed to differentiate viable cells from dead bacteria, thus providing a more accurate viability assessment. The established standard curve (y = 1.1716x + 3.1302; R^2^ = 0.9899) enabled precise conversion of cycle threshold (CT) values into viable bacterial counts (Fig. 3). Consistent with culture-based methods, PMA-qPCR data revealed a significant decline in *L. acidophilus* viability over time in all samples (Table 3), with the synbiotic SSL-FG 5% group retaining the highest viable counts during ripening (2.09 × 10^8^ CFU/g at day 15), reinforcing the notion of enhanced microbial stability via FG supplementation. These results are congruent with previous research employing PMA-qPCR for probiotic enumeration, such as García-Cayuela et al.^53^who validated the method’s specificity and reliability in fermented milk, and Desfossés-Foucault et al.^67^who documented similar viability patterns for *Lactobacillus* strains during cheddar cheese maturation.

The advantage of PMA-qPCR lies in its reduced time and higher sensitivity compared to conventional plate counting, while maintaining comparable accuracy, as demonstrated by Scariot et al.^54^. This molecular technique has also been effectively utilized for quantifying probiotic and pathogenic bacteria in diverse food matrices^68,69^. For instance, Do, Lao, and Le^70^ successfully applied PMA-qPCR to enumerate pathogenic bacteria in dairy products, highlighting its utility for live cell detection in complex microbiological assessments.

Overall, the integration of FG as a prebiotic ingredient in Iranian white cheese clearly contributed to the enhanced survival and persistence of *L. acidophilus*, suggesting promising implications for extending the functional shelf life and health benefits of synbiotic dairy products.

**Table 3:** Enumeration of viable (cfu/g) *L. acidophilus* cells in probiotic and synbiotic cheeses using the PMA method.

Sample	PSL	SSL-FG 2.5%	SSL-FG 5%
0 Day	1.15 × 10^9^	1.16 × 10^9^	1.16 × 10^9^
15 Day	2.88 × 10^7^	1.15 × 10^8^	2.09 × 10^8^
30 Day	2.51 × 10^6^	1.45 × 10^7^	2.96 × 10^7^
60 Day	1.35 × 10^6^	2.55 × 10^6^	1.55 × 10^7^

### Sensory properties

**Fig. 4:**
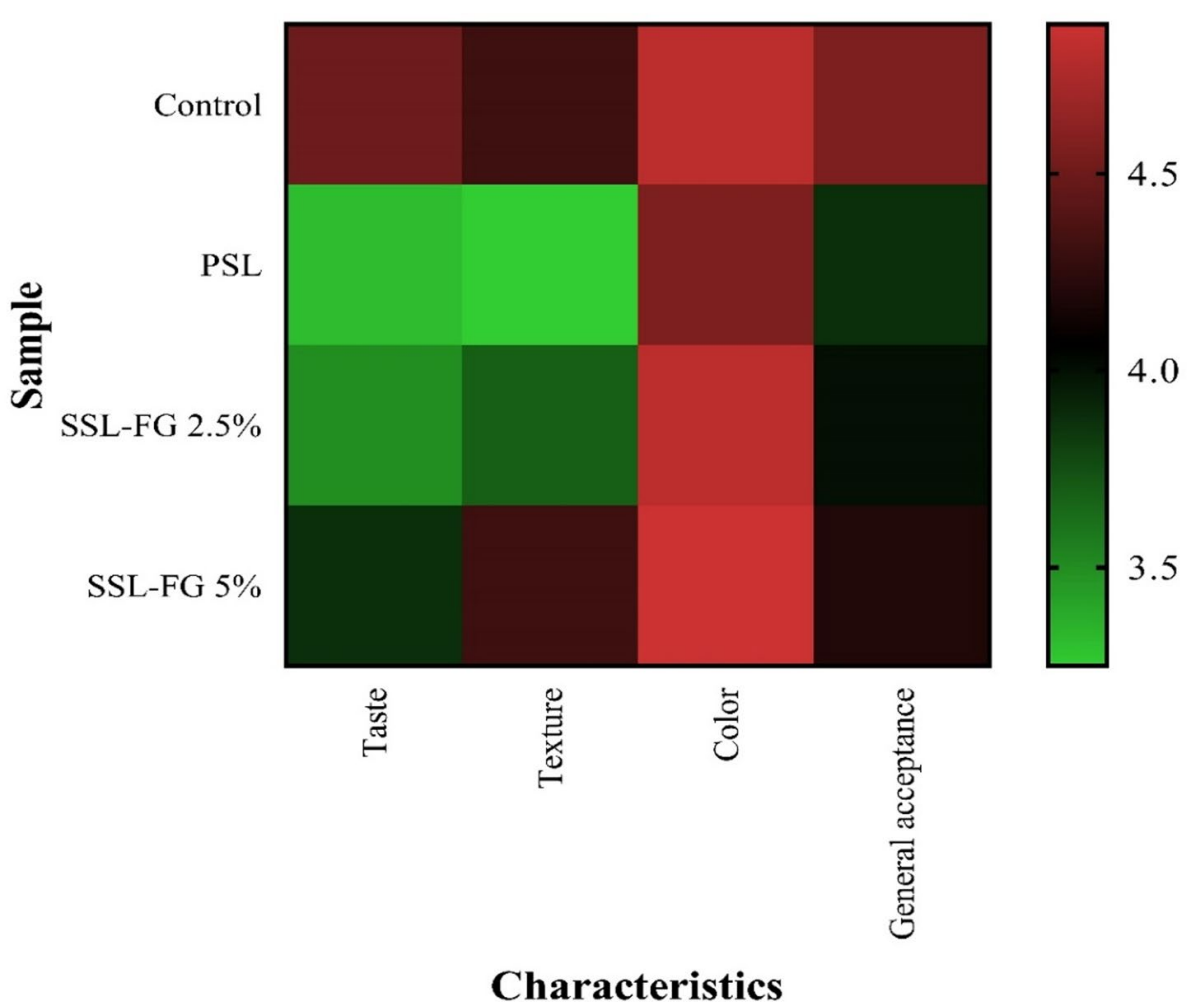
Comparison of sensory evaluation of cheeses prepared with FG and *L. acidophilus* using heat map analysis.

Appropriate sensory characteristics are crucial for the successful market acceptance of food products. Even nutritionally valuable items may fail commercially if they do not meet consumer preferences^71^. In this study, sensory evaluation of Iranian white cheese supplemented with FG at 2.5% and 5% concentrations (SSL-FG 2.5% and SSL-FG 5%) was performed using a five-point hedonic scale, assessing attributes including taste, texture, color, and overall acceptance. The results, visualized through a heat map (Fig. 4), demonstrated that the control cheese (without probiotic or FG) generally scored highest across sensory parameters, with scores of 4.50 for taste, 4.31 for texture, 4.81 for color, and 4.56 for overall acceptance. Among treated samples, the probiotic cheese without FG (PSL) received the lowest ratings, particularly in taste (3.31) and texture (3.25), contributing to a reduced overall acceptance score (3.88). Incorporation of FG at 2.5% slightly diminished some sensory attributes, notably color and overall acceptance, compared to the control. However, increasing the FG concentration to 5% ameliorated these effects, resulting in the highest color score (4.88) among all samples and an overall acceptance score (4.19) approaching that of the control. This indicates that higher levels of FG may help alleviate some sensory issues related to probiotic addition and enhance product appeal.

These findings align with those of Habibi and Jooyandeh^36^who reported that Persian gum enhanced sensory properties and increased general acceptance in Iranian white cheese, underscoring the potential of plant-derived gums to improve product quality. Conversely, studies involving *Lepidium perfoliatum* seed gum and FG as fat replacers in cream cheese revealed a general decline in sensory attributes over storage, including color and odor, with scores lower than those of high-fat controls^72^. Similarly, the addition of basil seed and xanthan gums was associated with reduced sensory scores compared to controls^73^. However, evidence also exists supporting the beneficial effects of FG on sensory quality, as demonstrated by Akl et al.^74^who reported improved sensory attributes in dairy products supplemented with FG. Taken together, these results highlight the complex interactions between prebiotic polysaccharides and sensory characteristics, which may vary depending on gum type, concentration, and product matrix, emphasizing the need for optimization to balance functional benefits with consumer acceptability.

### Texture properties

FG is not only known for its health benefits and nutritional value, but also for its gelling properties that help improve the texture of various food products, such as dairy items and cheeses. This functional polysaccharide enhances viscosity, primarily due to its arabinoxylan content, which consists of neutral, high molecular weight polysaccharides^11^. In the present study, variance analysis (Table 4) revealed significant effects (*p* < 0.01) of FG addition on key textural parameters of synbiotic Iranian white cheese. These parameters include springiness (mean square: 0.56 mm), cohesiveness (0.0043 N), and chewiness (21.393 mJ). Adhesion, however, showed no significant difference (0.0001 N.m), indicating random variation unaffected by treatments. Multi-domain mean comparisons showed that the probiotic cheese sample without FG (PSL) was different from the other groups. It had the highest adhesion (0.1029 N.m), although this difference was not statistically significant. In comparison, the commercial market sample (CMS) and the control group had the lowest adhesion values (0.0872 and 0.0871 N.m, respectively). Springiness of SSL-FG 5% (3.6333 mm) closely resembled control (3.7666 mm) and CMS (3.6333 mm) samples (*p* < 0.01). Cohesiveness was significantly higher in PSL (0.2501 N) than in SSL-FG 2.5% (0.1910 N), SSL-FG 5% (0.1930 N), control (0.1967 N), and CMS (0.1940 N) (*p* < 0.01). Likewise, chewiness values placed PSL (5.0275 mJ) as furthest from control (4.5666 mJ) and CMS (4.5451 mJ), while SSL-FG 5% (4.5167 mJ) and SSL-FG 2.5% (4.4667 mJ) were closest, indicating that FG addition preserves desirable textural properties. These findings support the use of FG for formulation optimization aiming to enhance cheese texture. Similar results were reported by Akl et al.^74^who found that FG, as a fat replacer in probiotic cream cheese, increased viscosity and synergistically improved sensory attributes, making it a suitable alternative for consumers restricting fat intake. Likewise, Bazezew et al.^75^ successfully employed *Ximenia americana* seed gum as a fat substitute in cheddar cheese without compromising texture or flavor. However, gum addition does not universally enhance texture; for example, Hesarinejad et al.^76^ demonstrated that gelatin and guar gum had no significant impact on ricotta cheese texture.

**Fig. 5:**
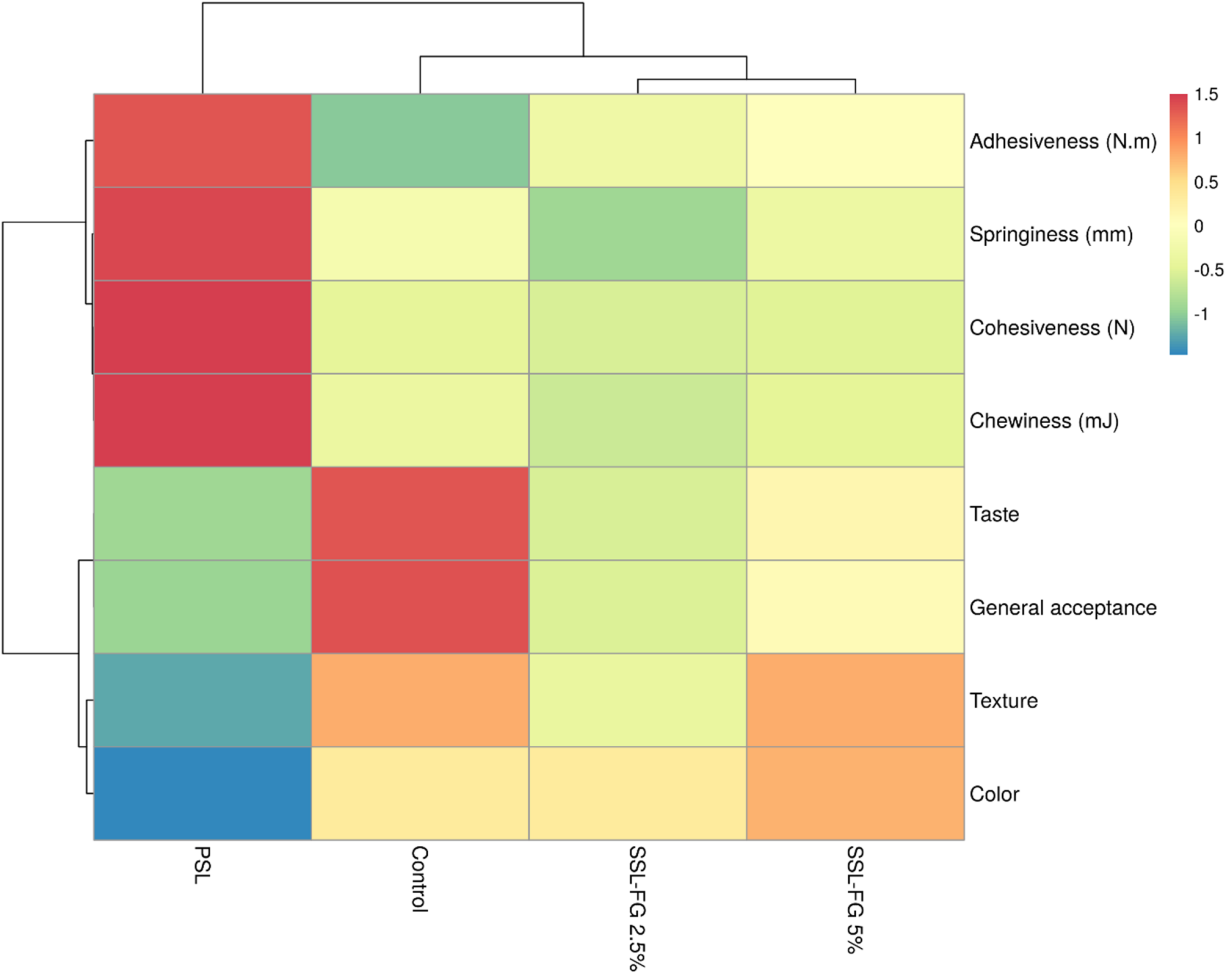
Cluster analysis for grouping produced cheeses and sensory-textural characteristics.

To comprehensively analyze the relationship between sensory and textural attributes, cluster analysis was performed, a statistical technique frequently utilized in dairy product characterization to group samples based on similarities across multiple attributes^77^. The cluster analysis results (Fig. 5) indicated that SSL-FG 2.5% and SSL-FG 5% samples clustered closely, reflecting high similarity in texture and color. SSL-FG 5% exhibiting notably higher scores, including general acceptance. The control sample joined this cluster but exhibited distinct differences, particularly in adhesiveness and taste, highlighting subtle yet important sensory-textural divergences. The PSL sample appeared as the most distant member, underscoring its significant divergence in these characteristics. The analysis further classified measured traits into two principal groups: the first comprising adhesiveness, cohesiveness, texture, and color, likely linked through molecular interactions within the cheese matrix, and the second including springiness, chewiness, taste, and general acceptance, reflecting the interplay between textural and sensory perception. This grouping suggests that FG addition contributes to a balanced enhancement of mechanical and sensory properties, ultimately improving consumer acceptance. Such applications of cluster analysis align with prior studies, such as de la Haba Ruiz et al.^78^who employed this method to group cheeses by physicochemical characteristics, and Bittante et al.^79^who used cluster and factor analyses on 1050 cheese varieties to reveal latent structures and facilitate classification based on multidimensional properties. Overall, these results emphasize cluster analysis as a powerful tool for elucidating complex relationships in food matrices and support the beneficial role of FG in improving the texture and sensory appeal of synbiotic Iranian white cheese.

**Table 4:** Variance analysis of textural characteristics of produced probiotic and synbiotic cheeses.

Sample	Texture characteristics at the Sixtieth day			
	Adhesiveness (*N*.m)	Springiness (mm)	Cohesiveness (*N*)	Chewiness (mJ)
Control	0.0887 ± 0.0089^ab^	3.7666 ± 0.0305^b^	0.1967 ± 0.0120^b^	4.5666 ± 0.5788^b^
CM_S_	0.0871 ± 0.0044^b^	3.68 ± 0.0692^c^	0.1940 ± 0.0112^b^	4.5451 ± 0.3312^c^
PSL	0.1029 ± 0.0055^a^	4.1733 ± 0.0702^a^	0.2501 ± 0.0018^a^	5.0275 ± 0.2492^a^
SSL-FG 2.5%	0.0920 ± 0.0103^ab^	3.4533 ± 0.1171^b^	0.1910 ± 0.0183^b^	4.4667 ± 0.6417^b^
SSL-FG 5%	0.0939 ± 0.0089^ab^	3.6333 ± 0.1942^b^	0.1930 ± 0.0017^b^	4.5167 ± 0.3871^b^
Mean square	0.0001^ns^	0.5600**	0.0043**	21.3930**

CMS: Commercial market sample.

**Significance (*p* < 0.01).

### Structural characterization

**Fig. 6:**
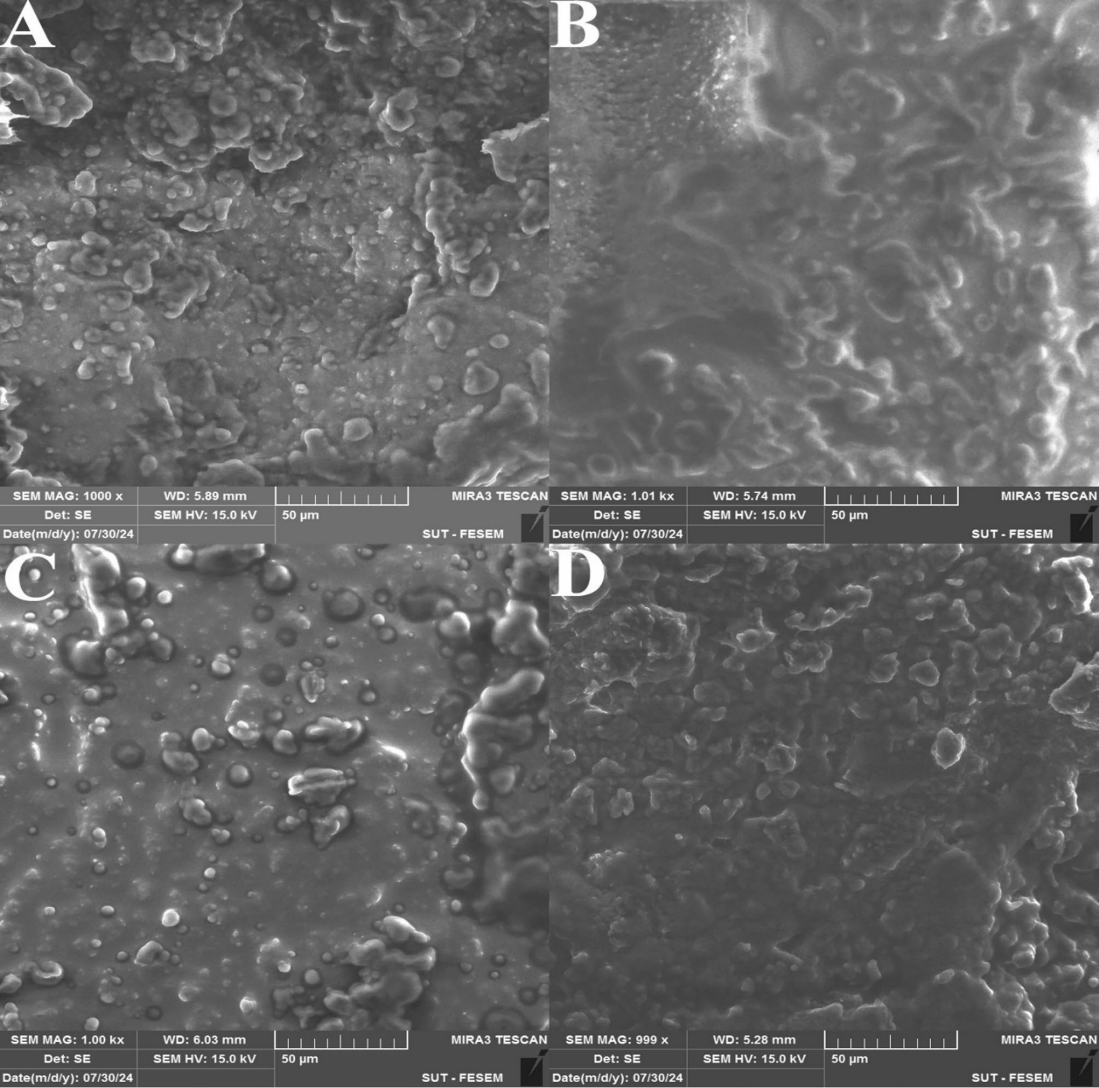
Microstructure of the cheese samples produced using SEM: Control (A), PSL (B), SSL-FG 2.5% (C), and SSL-FG 5% (D).

The microstructural characteristics of control, PSL, SSL-FG 2.5%, and SSL-FG 5% cheese samples were investigated using scanning electron microscopy (SEM), revealing notable differences aligned with textural assessments. As illustrated in Fig. 6A–D, the control cheese exhibited a denser and more compact matrix structure, whereas the PSL sample demonstrated a significantly reduced matrix density, resulting in a looser and more porous structure. This observation corroborates the texture analysis, where PSL deviated most from the control. The incorporation of FG notably enhanced matrix cohesion, likely due to its role as an active filler that disperses within the cheese matrix similarly to fat, disrupting casein bonds and creating a cream cheese-like texture with improved mouthfeel^72^. These findings align with Shahraki et al.^72^who reported similar microstructural improvements in low-fat probiotic cream cheese upon FG addition. Furthermore, Habibi and Jooyandeh^80^ demonstrated that Persian gum enhanced the texture of Iranian white cheese by promoting a more dispersed matrix structure, attributed to increased hydration capacity and consequent reduced compactness, consistent with our results.

**Fig. 7:**
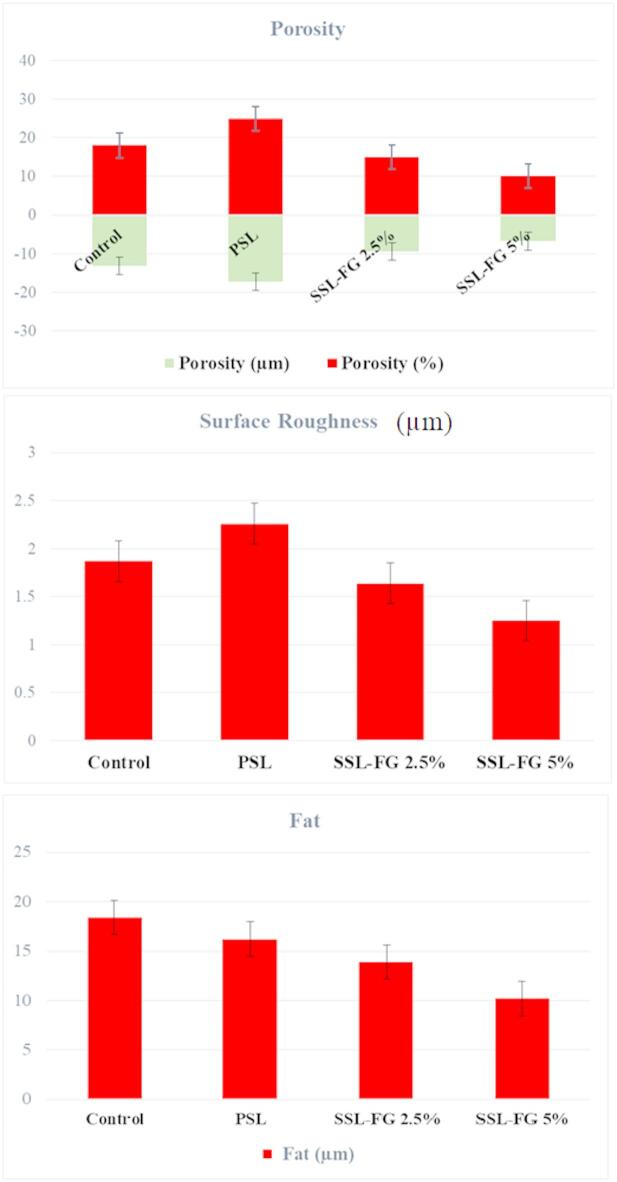
Structural characteristics of the cheeses sample produced: porosity (A), roughness (B), and fat (C) diameter.

Quantitative image analysis of cheese porosity, surface roughness, and fat globule diameter was conducted using ImageJ software (Fig. 7A–C), providing detailed microstructural metrics. The PSL sample exhibited significantly increased porosity both in size (µm) and percentage, exceeding that of the control, while FG supplementation reduced porosity closer to control levels; specifically, SSL-FG 2.5% and SSL-FG 5% samples showed porosity percentages of 9% and 7%, and pore sizes of 15 μm and 10 μm, respectively. Surface roughness measurements followed a similar trend, with PSL exhibiting the highest roughness diameter (2.25 μm), decreasing progressively with higher gum content 1.75 μm in control, 1.65 μm in SSL-FG 2.5%, and 1.25 μm in SSL-FG 5%. Fat globule diameter decreased from 18 μm in the control to 16 μm (PSL), 14 μm (SSL-FG 2.5%), and 10 μm (SSL-FG 5%). This reduction is attributed to the emulsifying properties of FG, which increases the viscosity of the aqueous phase and interacts with the protein network, forming a protective layer around fat globules that prevents coalescence and restricts their movement, thereby resulting in finer fat dispersion within the cheese matrix^81^.

The application of ImageJ for microscopic image analysis offers a robust, non-destructive quantitative method to evaluate cheese microstructure, including porosity, fat particle distribution, and protein network uniformity. This tool has been widely used across various cheese types to elucidate the impact of processing variables on texture and quality^82,83^. For instance, Wu et al.^84^ utilized ImageJ to assess porosity changes in cream cheese induced by calcium reduction, demonstrating a correlation between decreased porosity and softer cheese texture. These insights reinforce the utility of microstructural analysis in optimizing cheese formulation and highlight the beneficial role of FG in improving synbiotic Iranian white cheese by modulating its microstructure and consequently its sensory and textural properties.

## Conclusion

The production of synbiotic Iranian white cheese offers a valuable opportunity to improve consumer health through the daily intake of functional dairy products. This study demonstrated that incorporating of FG at 2.5% and especially 5% may significantly enhanced the expression of critical functional genes (*Aspartate kinase*, *SOS response-related peptidase*, *ROK family protein*, and *LytR-CpsA-Psr*) in *L. acidophilus*. This upregulation was closely associated with improved probiotic viability, metabolic activity, and microbial stability throughout the ripening period, particularly peaking at day 15 and gradually declining by day 60. These molecular changes translated into enhanced cheese texture, as evidenced by improved cohesiveness, springiness, and chewiness, as well as favorable microstructural modifications such as decreased porosity and fat globule size, which collectively contributed to a more stable and appealing product. Sensory evaluation further confirmed that the addition of FG positively influenced consumer acceptance, balancing functional benefits with desirable organoleptic properties. The application of PMA-qPCR for microbial quantification validated the superior survival of probiotic bacteria in FG -enriched samples, underscoring its protective role during storage. Notwithstanding the important contributions of this research, a number of limitations should be noted. The study was only undertaken in one strain of *L. acidophilus* because the study was originally designed to assess the initial impacts of FG and the nature of the nutritive components on gene expression and ccharacteristics of the cheese produced. While observations made may relate to other strains, testing several would require significant time and resources. In the sensory evaluation, a small panel with a 5-point scale was adopted due to limited access to consumers and study design practicality, which reduces the opportunity for consumer preference data granularity. Additionally, the mechanistic effect of FG on gene expression has yet to be fully revealed, and broader molecular studies, such as transcriptomic profiling, should be considered for further investigation.

Scaling such approaches could enhance the commercial viability of functional cheeses. This would contribute to public health promotion by delivering dairy products with prolonged shelf life, enhanced nutritional value, and improved sensory quality. Therefore, FG presents a promising natural ingredient for the design of next-generation synbiotic dairy products with significant health and economic benefits.

## Data Availability

The datasets generated and/or analyzed during the current study are available from the corresponding author on reasonable request. All methods were performed in accordance with relevant guidelines and standard protocols to ensure reproducibility.

## References

[1] Christaki, S., Moschakis, T., Kyriakoudi, A., Biliaderis, C. G. & Mourtzinos, I. Recent advances in plant essential oils and extracts: delivery systems and potential uses as preservatives and antioxidants in cheese. *Trends Food Sci. Technol.* **116**, 264–278 (2021).

[2] Kamath, R., Basak, S. & Gokhale, J. Recent trends in the development of healthy and functional cheese analogues-a review. *LWT* **155**, 112991 (2022).

[3] Tayebi-Moghaddam, S., Khatibi, R., Taklavi, S., Hosseini-Isfahani, M. & Rezaeinia, H. Sustained-release modeling of clove essential oil in Brine to improve the shelf life of Iranian white cheese by bioactive electrospun Zein. *Int. J. Food Microbiol.* **355**, 109337 (2021). 34340156 10.1016/j.ijfoodmicro.2021.109337

[4] Bakhtiyari, M., Hamidi-Esfahani, Z. & Barzegar, M. The influence of co-encapsulated L. plantarum and Silybum Marianum seed extract on the physicochemical properties of synbiotic cheese during ripening. *Food Chemistry: X*. **23**, 101674 (2024). 39139494 10.1016/j.fochx.2024.101674 PMC11321426

[5] Ryan, J. et al. Microbial, physico-chemical and sensory characteristics of Mango juice‐enriched probiotic dairy drinks. *Int. J. Dairy Technol.* **73**, 182–190 (2020).

[6] María Remes-Troche, J. et al. *Lactobacillus acidophilus* LB: A useful pharmabiotic for the treatment of digestive disorders. *Therapeutic Adv. Gastroenterol.* **13**, 1756284820971201 (2020). 10.1177/1756284820971201 PMC7692339 33281937

[7] Sharma, M., Wasan, A. & Sharma, R. K. Recent developments in probiotics: an emphasis on bifidobacterium. *Food Bioscience*. **41**, 100993 (2021).

[8] Oeffner, S. et al. Effect of flaxseed supplementation rate and processing on the production, fatty acid profile, and texture of milk, butter, and cheese. *J. Dairy Sci.* **96**, 1177–1188 (2013). 23219126 10.3168/jds.2012-5941

[9] Luo, J. et al. Flaxseed gum reduces body weight by regulating gut microbiota. *J. Funct. Foods.* **47**, 136–142 (2018).

[10] Liu, J., Shim, Y. Y., Timothy, J. T., Wang, Y. & Reaney, M. J. Flaxseed gum a versatile natural hydrocolloid for food and non-food applications. *Trends Food Sci. Technol.* **75**, 146–157 (2018).

[11] Lorenc, F., Jarošová, M., Bedrníček, J., Smetana, P. & Bárta, J. Structural characterization and functional properties of flaxseed hydrocolloids and their application. *Foods* **11**, 2304 (2022). 35954070 10.3390/foods11152304 PMC9368198

[12] Zrimec, J., Buric, F., Kokina, M., Garcia, V. & Zelezniak, A. Learning the regulatory code of gene expression. *Front. Mol. Biosci.* **8**, 673363 (2021). 34179082 10.3389/fmolb.2021.673363 PMC8223075

[13] Buccitelli, C. & Selbach, M. mRNAs, proteins and the emerging principles of gene expression control. *Nat. Rev. Genet.* **21**, 630–644 (2020). 32709985 10.1038/s41576-020-0258-4

[14] García-Burgos, M., Moreno-Fernández, J., Alférez, M. J., Díaz-Castro, J. & López-Aliaga, I. New perspectives in fermented dairy products and their health relevance. *J. Funct. Foods.* **72**, 104059 (2020).

[15] King, R. C. *Handbook of genetics: volume 1 bacteria, bacteriophages, and fungi* Vol. 1 (Springer Science & Business Media, 2013).

[16] Selwood, T. & Jaffe, E. K. Dynamic dissociating homo-oligomers and the control of protein function. *Arch. Biochem. Biophys.* **519**, 131–143 (2012). 22182754 10.1016/j.abb.2011.11.020 PMC3298769

[17] Viola, R. E. The central enzymes of the aspartate family of amino acid biosynthesis. *Acc. Chem. Res.* **34**, 339–349 (2001). 11352712 10.1021/ar000057q

[18] Rafalski, J. A. & Falco, S. C. Structure of the yeast HOM3 gene which encodes aspartokinase. *J. Biol. Chem.* **263**, 2146–2151 (1988). 2892836

[19] Polosina, Y. (Elsevier, (2014).

[20] Thompson, P. S., Amidon, K. M., Mohni, K. N., Cortez, D. & Eichman, B. F. Protection of Abasic sites during DNA replication by a stable Thiazolidine protein-DNA cross-link. *Nat. Struct. Mol. Biol.* **26**, 613–618 (2019). 31235915 10.1038/s41594-019-0255-5 PMC6628887

[21] Shukla, V. et al. HMCES functions in the alternative end-joining pathway of the DNA DSB repair during class switch recombination in B cells. *Mol. Cell*. **77**, 384–394 (2020). e384. 31806351 10.1016/j.molcel.2019.10.031 PMC6980713

[22] Titgemeyer, F., Reizer, J., Reizer, A. & Saier, M. H. Jr Evolutionary relationships between sugar kinases and transcriptional repressors in bacteria. *Microbiology* **140**, 2349–2354 (1994). 7952186 10.1099/13500872-140-9-2349

[23] Ruiz-Villafán, B., Rocha, D., Romero, A. & Sánchez, S. Relevance of microbial glucokinases. *Biotechnol. Microb. Enzy.* **25**, 249–278 (2023).

[24] Wen, S., Feng, D., Chen, D., Yang, L. & Xu, Z. Molecular epidemiology and evolution of haemophilus influenzae. *Infect. Genet. Evol.* **80**, 104205 (2020). 31981610 10.1016/j.meegid.2020.104205

[25] Denamur, E., Clermont, O., Bonacorsi, S. & Gordon, D. The population genetics of pathogenic Escherichia coli. *Nat. Rev. Microbiol.* **19**, 37–54 (2021). 32826992 10.1038/s41579-020-0416-x

[26] Michels, R., Last, K., Becker, S. L. & Papan, C. Update on coagulase-negative staphylococci—what the clinician should know. *Microorganisms* **9**, 830 (2021). 33919781 10.3390/microorganisms9040830 PMC8070739

[27] Kawai, Y. et al. A widespread family of bacterial cell wall assembly proteins. *EMBO J.* **30**, 4931–4941 (2011). 21964069 10.1038/emboj.2011.358 PMC3243631

[28] Baumgart, M., Schubert, K., Bramkamp, M. & Frunzke, J. Impact of LytR-CpsA-Psr proteins on cell wall biosynthesis in Corynebacterium glutamicum. *J. Bacteriol.* **198**, 3045–3059 (2016). 27551018 10.1128/JB.00406-16 PMC5075034

[29] Sungatullina, A., Petrova, T., Kharina, M., Mikshina, P. & Nikitina, E. Effect of flaxseed mucilage on the probiotic, antioxidant, and structural-mechanical properties of the different Lactobacillus cells. *Fermentation* **9**, 486 (2023).

[30] Qian, K., Cui, S., Wu, Y. & Goff, H. Flaxseed gum from flaxseed hulls: extraction, fractionation, and characterization. *Food Hydrocoll.* **28**, 275–283 (2012).

[31] Kaushik, P., Dowling, K., Adhikari, R., Barrow, C. J. & Adhikari, B. Effect of extraction temperature on composition, structure and functional properties of flaxseed gum. *Food Chem.* **215**, 333–340 (2017). 27542483 10.1016/j.foodchem.2016.07.137

[32] Mojaddar Langroodi, A., Mehdizadeh, T., Majidi, L. & Neyriz-Naghadehi, M. *Lactobacillus acidophilus* and anethum graveolens essential oil in Iranian cheese against Escherichia coli O157: H7. *Flavour Fragr. J.* **36**, 190–196 (2021).

[33] Padilha, M., Morales, M. L. V., Vieira, A. D. S., Costa, M. G. M. & Saad, S. M. I. A prebiotic mixture improved *Lactobacillus acidophilus* and bifidobacterium animalis Gastrointestinal in vitro resistance in petit-suisse. *Food Funct.* **7**, 2312–2319 (2016). 27112363 10.1039/c5fo01592h

[34] Mehdizadeh, T., Razavi, M. & Esmaeili Koutamehr, M. The effect of wild Leek (Allium ampeloprasum) on growth and survival of *Lactobacillus acidophilus* and sensory properties in Iranian white cheese. *Res. Innov. Food Sci. Technol.* **7**, 431–444 (2019).

[35] Mirzaei, H., Pourjafar, H. & Homayouni, A. Effect of calcium alginate and resistant starch microencapsulation on the survival rate of *Lactobacillus acidophilus* La5 and sensory properties in Iranian white Brined cheese. *Food Chem.* **132**, 1966–1970 (2012).

[36] Jooyandeh, H., Goudarzi, M., Rostamabadi, H. & Hojjati, M. Effect of Persian and almond gums as fat replacers on the physicochemical, rheological, and microstructural attributes of low-fat Iranian white cheese. *Food Sci. Nutr.* **5**, 669–677 (2017). 28572956 10.1002/fsn3.446 PMC5448388

[37] Gholamhosseinpour, A. & Zare, S. Investigating the effect of Persian gum on physicochemical, textural and rheological characteristics of UF white Brined cheese during ripening. *Int. Dairy J.* **155**, 105952 (2024).

[38] Garenne, D. et al. Cell-free gene expression. *Nat. Reviews Methods Primers*. **1**, 49 (2021).

[39] Saber, A., Alipour, B., Faghfoori, Z. & Khosroushahi, A. Y. Secretion metabolites of dairy Kluyveromyces Marxianus AS41 isolated as probiotic, induces apoptosis in different human cancer cell lines and exhibit anti-pathogenic effects. *J. Funct. Foods.* **34**, 408–421 (2017).

[40] Viçosa, G. N. et al. Impact of co-cultivation with Enterococcus faecalis over growth, enterotoxin production and gene expression of Staphylococcus aureus in broth and fresh cheeses. *Int. J. Food Microbiol.* **308**, 108291 (2019). 31437692 10.1016/j.ijfoodmicro.2019.108291

[41] Casquete, R., Benito, M. J., Aranda, E., Martín, A. & Ruiz-Moyano, S. De Guía córdoba, M. Gene expression of Aspergillus flavus strains on a cheese model system to control aflatoxin production. *J. Dairy Sci.* **102**, 7765–7772 (2019). 31301828 10.3168/jds.2019-16722

[42] Homayouni-Tabrizi, M., Asoodeh, A. & Soltani, M. Cytotoxic and antioxidant capacity of camel milk peptides: effects of isolated peptide on superoxide dismutase and catalase gene expression. *J. Food Drug Anal.* **25**, 567–575 (2017). 28911643 10.1016/j.jfda.2016.10.014 PMC9328817

[43] Ali, M. A. et al. Development of a standard curve to account for viable loads of bifidobacterium animalis subsp. Lactis HN019 using RNA by real-time PCR. *J. AOAC Int.* **102**, 532–538 (2019). 30135002 10.5740/jaoacint.18-0216

[44] Dolan, A., Burgess, C., Fanning, S. & Duffy, G. Application of quantitative reverse-transcription PCR (qRT‐PCR) for the determination of the total viable count (TVC) on meat samples. *J. Appl. Microbiol.* **109**, 91–98 (2010). 20028440 10.1111/j.1365-2672.2009.04636.x

[45] Masco, L., Vanhoutte, T., Temmerman, R., Swings, J. & Huys, G. Evaluation of real-time PCR targeting the 16S rRNA and RecA genes for the enumeration of bifidobacteria in probiotic products. *Int. J. Food Microbiol.* **113**, 351–357 (2007). 17088006 10.1016/j.ijfoodmicro.2006.07.021

[46] Ahmed, H. A., Tahoun, A. B., Elez, A., El-Hamid, R. M. A., Abd Ellatif, S. S. & M. I. & Prevalence of yersinia Enterocolitica in milk and dairy products and the effects of storage temperatures on survival and virulence gene expression. *Int. Dairy J.* **94**, 16–21 (2019).

[47] Torabi, F., Jooyandeh, H. & Noshad, M. Evaluation of physicochemical, rheological, microstructural, and microbial characteristics of synbiotic ultrafiltrated white cheese treated with transglutaminase. *J. Food Process. Preserv.* **45**, e15572 (2021).

[48] Atik, D. S. & Çoşkun, F. Some properties of probiotic yoghurt produced for babies by adding fruit puree, containing B. infantis, B. bifidum, B. longum, L. paracasei. *Turkish J. Agriculture-Food Sci. Technol.* **9**, 1840–1848 (2021).

[49] Rhoades, J. et al. Microbiological analysis of Greek protected designation of origin cheeses and characterisation of the isolated lactic acid bacteria. *Int. Dairy J.* **123**, 105183 (2021).

[50] Ong, L., Henriksson, A. & Shah, N. Development of probiotic cheddar cheese containing *Lactobacillus acidophilus*, lb. casei, lb. paracasei And bifidobacterium spp. And the influence of these bacteria on proteolytic patterns And production of organic acid. *Int. Dairy J.* **16**, 446–456 (2006).

[51] Buran, İ., Akal, C., Ozturkoglu-Budak, S. & Yetisemiyen, A. Rheological, sensorial and volatile profiles of synbiotic Kefirs produced from cow and goat milk containing varied probiotics in combination with fructooligosaccharide. *LWT* **148**, 111591 (2021).

[52] Fan, X. et al. A novel qPCR method for the detection of lactic acid bacteria in fermented milk. *Foods* **10**, 3066 (2021). 34945617 10.3390/foods10123066 PMC8700909

[53] Desfossés-Foucault, É. et al. Assessment of probiotic viability during cheddar cheese manufacture and ripening using Propidium monoazide-PCR quantification. *Front. Microbiol.* **3**, 350 (2012). 23060868 10.3389/fmicb.2012.00350 PMC3463833

[54] Scariot, M. C., Venturelli, G. L., Prudêncio, E. S. & Arisi, A. C. M. Quantification of Lactobacillus paracasei viable cells in probiotic yoghurt by Propidium monoazide combined with quantitative PCR. *Int. J. Food Microbiol.* **264**, 1–7 (2018). 29073460 10.1016/j.ijfoodmicro.2017.10.021

[55] Shi, Z. et al. PMA-qPCR method for the selective quantitation of viable lactic acid bacteria in fermented milk. *Front. Microbiol.* **13**, 984506 (2022). 36160254 10.3389/fmicb.2022.984506 PMC9491339

[56] Rugji, J. & Dinçoğlu, A. H. Biocontrol of Listeria monocytogenes by Bacillus coagulans GBI-30, 6086 in a synbiotic white Brined cheese: an in vitro model study. *LWT* **169**, 113982 (2022).

[57] Hurtado-Romero, A. et al. Utilization of blueberry-based ingredients for formulating a synbiotic Petit Suisse cheese: physicochemical, microbiological, sensory, and functional characterization during cold storage. *LWT* **183**, 114955 (2023).

[58] Chaharaein, M., Sadeghi, E., Mohammadi, R., Rouhi, M. & Soltani, M. The effect of β-glucan and inulin on the reduction of aflatoxin B1 level and assessment of textural and sensory properties in chicken sausages. *Curr. Res. Food Sci.* **4**, 765–772 (2021). 34766007 10.1016/j.crfs.2021.10.007 PMC8569632

[59] Shehata, M. G., El-Aziz, A., Darwish, N. M., El-Sohaimy, S. A. & A. G. & Lacticaseibacillus paracasei KC39 immobilized on prebiotic wheat Bran to manufacture functional soft white cheese. *Fermentation* **8**, 496 (2022).

[60] Darnay, L. et al. Comparison of different visual methods to follow the effect of milk heat treatment and MTGase on appearance of semi-hard Buffalo cheese. *Food Control*. **139**, 109049 (2022).

[61] Ross, M. M. et al. Parameters affecting the printability of 3D-printed processed cheese. *Innovative Food Sci. Emerg. Technol.* **72**, 102730 (2021).

[62] Khedr, M. et al. FolE gene expression for folic acid productivity from optimized and characterized probiotic Lactobacillus delbrueckii. *J. Genetic Eng. Biotechnol.* **21**, 169 (2023). 10.1186/s43141-023-00603-9 PMC10728034 38108957

[63] Mancini, A. et al. In vitro probiotic characterization of high GABA producing strain lactobacilluas brevis DSM 32386 isolated from traditional wild alpine cheese. *Ann. Microbiol.* **69**, 1435–1443 (2019).

[64] Dishan, A. & Gönülalan, Z. Lacticaseibacillus paracasei AD22 stress response in Brined white cheese matrix: in vitro probiotic profiles and molecular characterization. *Probiotics Antimicrob. Proteins*, 1–14 (2024). 10.1007/s12602-024-10216-4 PMC12055941 38421575

[65] Noutsopoulos, D. et al. NisA gene expression, and in situ activity of novel Lactococcus lactis subsp. Cremoris costarter culture in commercial hard cheese production. *J. Food. Prot.* **80**, 2137–2146 (2017). 29182362 10.4315/0362-028X.JFP-17-245

[66] Alhssan, E., Ercan, S. Ş. & Bozkurt, H. Effect of flaxseed mucilage and gum Arabic on probiotic survival and quality of Kefir during cold storage. *Foods* **12**, 662 (2023). 36766188 10.3390/foods12030662 PMC9914877

[67] García-Cayuela, T., Tabasco, R., Peláez, C. & Requena, T. Simultaneous detection and enumeration of viable lactic acid bacteria and bifidobacteria in fermented milk by using Propidium monoazide and real-time PCR. *Int. Dairy J.* **19**, 405–409 (2009).

[68] Guo, L. et al. Identification and quantification of viable Lacticaseibacillus rhamnosus in probiotics using validated PMA-qPCR method. *Front. Microbiol.* **15**, 1341884 (2024). 38298895 10.3389/fmicb.2024.1341884 PMC10828034

[69] Petersen, M., Ma, L. & Lu, X. Rapid determination of viable but non-culturable Campylobacter jejuni in food products by loop-mediated isothermal amplification coupling Propidium monoazide treatment. *Int. J. Food Microbiol.* **351**, 109263 (2021). 34116344 10.1016/j.ijfoodmicro.2021.109263

[70] Do, T., Lao, T. D. & Le, T. Establishment of PMA Real-Time PCR method to detect viable cells of Listeria monocytogenes and Salmonella spp. In milk and dairy products. *Asian J. Pharm. Res. Health Care*. **20**, 146–156 (2021).

[71] Rosa, M. C. et al. Dairy products with prebiotics: an overview of the health benefits, technological and sensory properties. *Int. Dairy J.* **117**, 105009 (2021).

[72] Shahraki, R., Elhamirad, A. H., Hesari, J., Noghabi, M. S. & Nia, A. P. A low-fat synbiotic cream cheese containing herbal gums, bifidobacterium adolescentis and Lactobacillus rhamnosus: physicochemical, rheological, sensory, and microstructural characterization during storage. *Food Sci. Nutr.* **11**, 8112–8120 (2023). 38107124 10.1002/fsn3.3731 PMC10724580

[73] Portaghi, J., Heshmati, A., Taheri, M., Ahmadi, E. & Khaneghah, A. M. Effect of Basil seed and Xanthan gum on physicochemical, textural, and sensory characteristics of low-fat cream cheese. *Food Sci. Nutr.* **11**, 6060–6072 (2023). 37823144 10.1002/fsn3.3542 PMC10563744

[74] Akl, E. M., Abdelhamid, S. M., Wagdy, S. M. & Salama, H. H. Manufacture of functional fat-free cream cheese fortified with probiotic bacteria and flaxseed mucilage as a fat replacing agent. *Curr. Nutr. Food Sci.* **16**, 1393–1403 (2020).

[75] Bazezew, A. M. et al. Application of Ximenia Americana seed mucilage as a fat substitute to enhance the characteristics of low-fat cheddar cheese. *J. Food Meas. Charact.* **19**, 630–643 (2025).

[76] Hesarinejad, M. A., Lorenzo, J. M. & Rafe, A. Influence of gelatin/guar gum mixture on the rheological and textural properties of restructured Ricotta cheese. *Carbohydr. Polym. Technol. Appl.* **2**, 100162 (2021).

[77] Eroglu, A., Dogan, M., Toker, O. S. & Yilmaz, M. T. Classification of Kashar cheeses based on their chemical, color and instrumental textural characteristics using principal component and hierarchical cluster analysis. *Int. J. Food Prop.* **18**, 909–921 (2015).

[78] de la Haba Ruiz, M. A., Pérez-Cacho, R., Dios Palomares, P., Galán-Soldevilla, H. & R. & Classification of artisanal Andalusian cheeses on physicochemical parameters applying multivariate statistical techniques. *Dairy. Sci. Technol.* **96**, 95–106 (2016).

[79] Bittante, G., Amalfitano, N., Ferragina, A., Lombardi, A. & Tagliapietra, F. Interrelationships among physical and chemical traits of cheese: explanatory latent factors and clustering of 37 categories of cheeses. *J. Dairy Sci.* **107**, 1980–1992 (2024). 37949396 10.3168/jds.2023-23538

[80] Habibi, S. A. & Jooyandeh, H. Investigation on the effect of Persian gum and transglutaminase enzyme on some physicochemical and microstructural characteristics of Low-Fat ultrafiltrated Iranian white cheese. *Food Sci. Nutr.* **12**, 9810–9821 (2024). 39619985 10.1002/fsn3.4551 PMC11606844

[81] Soleimani-Rambod, A., Zomorodi, S., Naghizadeh Raeisi, S., Khosrowshahi Asl, A. & Shahidi, S. A. The effect of Xanthan gum and flaxseed mucilage as edible coatings in cheddar cheese during ripening. *Coatings* **8**, 80 (2018).

[82] Bosakova-Ardenska, A. Recent trends in computer vision for cheese quality evaluation. *Eng. Proc.* **60**, 12 (2024).

[83] Lukinac, J., Jukić, M., Mastanjević, K. & Lučan, M. Application of computer vision and image analysis method in cheese-quality evaluation: a review. *Ukrain. Food J.* **36**, 192–214 (2018).

[84] Wu, Q. et al. Modulation of cream cheese physicochemical and functional properties with ultrafiltration and calcium reduction. *Food Chem.* **457**, 140010 (2024). 38908254 10.1016/j.foodchem.2024.140010

